# Effect of Heat Treatment on Microstructure and Properties Evolution of Stainless Steel Cladding Plate

**DOI:** 10.3390/ma16134809

**Published:** 2023-07-04

**Authors:** Luyan Li, Boshen Zhao, Yongtong Chen, Yi Ding

**Affiliations:** 1 College of Materials Science and Engineering, Nanjing Tech University, Nanjing 211800, China; 2 Shangi Institute for Advanced Materials Co., Ltd., Nanjing 210000, China

**Keywords:** stainless steel cladding plate, carbon diffusion, microstructure, mechanical property, corrosion resistance

## Abstract

Heat treatments are necessary sometimes in order to improve comprehensive properties of stainless steel cladding plate (SSCP). However, carbon atoms in carbon steel diffuse into stainless cladding during the heat treatment process, thus decreasing its corrosion resistance. In this paper, optical microscopy, scanning electron microscopy, and microhardness and shear testing were employed to characterize the microstructure and mechanical properties of the bonding interface in SSCP. Then, the corrosion resistance of the stainless steel cladding surface was evaluated by electrochemical tests. The results showed that the diffusion of carbon atoms played an important role in enhancing the bonding strength of SSCP, but might lead to intergranular sensitization of the cladding surface because of chromium carbide precipitation. Notably, this precipitation could be induced by quenching and tempering treatment, and hindered by solution treatment. Hence, the cladding surface on SSCP after single solution treatment possessed the superior corrosion resistance, and SSCP with continuous solution and tempering treatment exhibited the highest bonding strength.

## 1. Introduction

Stainless steel cladding plate (SSCP), composed of a carbon steel (CS) base and stainless steel (SS) cladding, possesses excellent mechanical and corrosion resistance properties [1] and has been widely used in oil, automobile, marine, and chemistry fields, among others [2,3]. Nowadays, the preparation method of large-scale industrialized SSCP is mainly hot-rolling bonding, due to its high efficiency, low cost, and simple operation [4].

However, the difference in the components between the SS cladding and CS base metal might lead to the interdiffusion of constituent elements under high-temperature and rolling conditions [5]. Diffusion of carbon atoms to carbon-less SS cladding from the carbon-rich CS base has been the focus of research. Interestingly, the diffusion of carbon atoms may improve the mechanical properties of SS cladding. Wu et al. [6] found that the increase in carbon content in the CS side lengthened the distance of carbon diffusion in SS cladding, thus improving its tensile and yield strength. However, due to the excessive carbide precipitation, elongation decreased. Liu et al. [7] found that the generation of interfacial oxides hindered carbon diffusion. With the decrease in oxides, carbon diffusion distance and interfacial bonding strength increased. According to Xiao et al. [8], the Ni interlayer could inhibit diffusion of carbon atoms and improve corrosion resistance of stainless steel cladding plate. However, due to the intrinsic low strength of the Ni interlayer, shear strength was reduced to some extent [9]. Though the carbon diffusion behavior plays a certain role in improving some mechanical properties, it will have a negative impact on the corrosion resistance of SS cladding. During hot-rolling bonding, precipitation of elements such as chromium carbide occurred at the austenitic grain boundary, leading to the intercrystalline sensitization of SS cladding. Therefore, the impact of carbon diffusion behavior is worthy of in-depth study to improve the mechanical strength and corrosion resistance of SSCP structures.

Heat treatment is a common process for improving bimetallic composite plate properties after hot rolling to meet the requirements of different cases [10,11]. Obviously, the heat treatment process will have a further impact on the diffusion of carbon elements, thereby affecting the corrosion resistance and mechanical strength of composite plate [12]. Ren et al. [13] investigated the effect of annealing temperature on copper/304 composite plates. With the increase in annealing temperature, the element diffusion distance increased, resulting in an increase in ductility but a decrease in the general strength and hardness. Jin et al. [14] studied the effect of heat treatments on 316L/Q345R composite plates. Results showed that the microstructure of specimens after quenching mainly consisted of martensite, with increased strength and poor ductility. Ma et al. [15] found that carbon diffusion distance decreased as quenching temperature increased, and a barely carburized layer was found near the 304/Q235 bonding interface at 1100 °C quenching. Hyojin [16] compared the effects of normalizing and tempering processes on the properties of the S32750 steel + EH40 composite plate for hull structures. The tempered state was more conducive to improving the tensile strength and yield strength than the normalized state.

With increasing demand for better performance of composite materials, heat treatment technology is continuously developing, and to date a variety of heat treatment methods have been derived [4,17]. However, research on the corrosion resistance and mechanical properties of SSCP under different heat treatment methods is still in its infancy. For 45# carbon steel, it is generally necessary to obtain good comprehensive mechanical properties by quenching and tempering treatment. The purpose of quenching at 850 °C is intended to transform the steel into a martensitic structure, which is then combined with tempering at different temperatures to substantially improve the mechanical properties of steel. However, quenching and tempering treatment promotes the diffusion of carbon atoms to stainless steel. Furthermore, the quenching and tempering temperatures are within the intergranular sensitization temperature range of 304 stainless steel, so precipitates existed. Therefore, the purpose of the second heat treatment (SSCP-HQT) in this paper was to study the effect on the corrosion resistance of stainless steel cladding based on the improvement in the mechanical properties of the carbon steel base. The solution treatment is followed by quenching in water to avoid any precipitation during cooling. Solution treatment was aimed to improve the corrosion resistance of SS cladding. Solution + tempering treatment was designed to improve the mechanical properties on the basis of corrosion resistance.

In order to achieve comprehensive improvement in corrosion resistance of SS cladding and mechanical properties of the CS base through heat treatment, in this study, three different heat treatment methods (quenching and tempering treatment, solution treatment, and solution treatment followed by tempering) were selected. In addition, the effect of carbide precipitation on microstructure evolution, corrosion resistance, and mechanical properties of the material was studied. This paper hopes to provide a theoretical basis for the practical application of stainless steel cladding plates.

## 2. Materials and Methods

### 2.1. Materials and Preparation of Heat Treatment

SSCP was bonded by hot rolling with 45 steel (Fujian, China) base and 304 stainless steel (Fujian, China) cladding. The chemical compositions of raw materials and heat treatment processes are listed in Table 1 and Table 2, respectively.

### 2.2. Experimental Methods

#### 2.2.1. Microstructural Characterization

After the SSCP specimen was ground and polished, the CS base was etched with 4 vol.% nital while SS cladding was exposed by electrolytic etching in 10% oxalic acid (Jiangsu, China) at 7 V for 40 s. A detailed study of the microstructure was carried out by optical microscopy (OM, Oberkochen, Germany). Scanning electron microscopy (SEM, SU5000) and energy dispersive X-ray spectroscopy (EDS, Oxford, UK) were used to characterize surface morphology and element distribution.

#### 2.2.2. Electrochemical Properties

Specimens with dimensions of 10 mm × 10 mm × 5 mm were produced for electrochemical tests of SS cladding on SSCP. Specimens were packed into epoxy resin and then ground with sandpaper in turn. All electrochemical tests were performed with a CHI760E (Shanghai, China) electrochemical workstation.

DL-EPR testing is widely used to quantitatively assess intergranular corrosion (IGC) susceptibility on the SS cladding surface. The test solution was 0.5 mol/L H_2_SO_4_ (Shanghai, China) + 0.01 mol/L KSCN (Shanghai, China) and the scanning rate was 1.5 mV/s. Before each electrochemical test, the specimen was immersed in the test solution for 1 h to obtain a stable state. Specimens were polarized from a potential of −0.45 (vs. SCE) and the scanning direction was reversed when the potential reached +0.3 V at the same scanning rate. 

The anodic polarization and electrochemical impedance spectroscopy (EIS) tests were carried out in 3.5 wt.% NaCl (Shanghai, China) at 25 ± 1 °C. Before each test, the specimen was immersed for 1 h to obtain a stable state. EIS was carried out at OCP with a perturbation amplitude of 10 mV, and a frequency range of 10^5^ HZ to 10^−2^ HZ. The anodic polarization curves were detected at a range of −0.3 V to +0.5 V (vs. OCP) at 1 mV/s.

#### 2.2.3. Mechanical Performance

The microhardness test was performed on HV-1000 (Guangdong, China) with a test force of 100 N and a hold time of 15 s. Measurements were made from the SS cladding to the CS base with an interval of 50 μm. The experiment was repeated to obtain the mapping of the microhardness.

**Figure 1:**
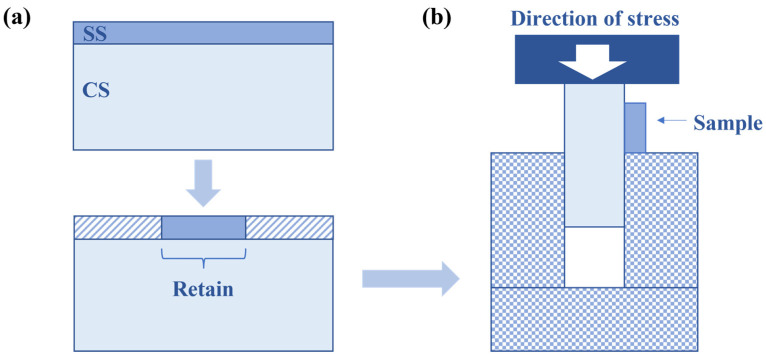
Schematic of shear test: (**a**) sample preparation; (**b**) testing device.

To evaluate mechanical properties, a shear test was carried out at a rate of 0.5 mm/min. Since the thickness of the SS cladding layer was 0.5 mm, the shear specimen was processed as shown in Figure 1a.

## 3. Results and Discussion

### 3.1. Microstructure Characterization

**Figure 2:**
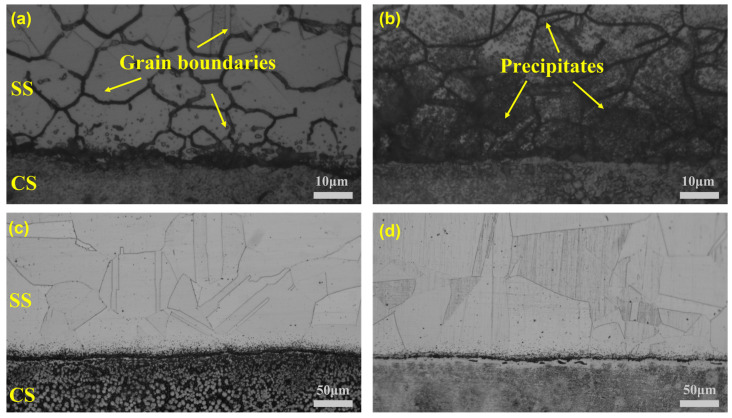
Microstructure of specimens: (**a**) SSCP-H, (**b**) SSCP-HQT, (**c**) SSCP-HS, and (**d**) SSCP-HST after electrochemical etching in 10% oxalic acid.

Figure 2 depicts the microstructure of specimens with various heat treatment conditions. A typical reticulated structure can be observed on SS cladding in SSCP-H (Figure 2a) because carbide precipitation in grain boundaries was eroded away after electrolytic erosion [18]. Additionally, the promotion of heat treatment to carbon atom diffusion was dominant, such as in SSCP-HQT shown in Figure 2b. A large number of both intragranular and intergranular carbide precipitations were detected in 304 SS cladding. It was noteworthy that few carbides precipitated in the SS cladding of SSCP-HS and SSCP-HST, where austenitic grain boundaries could be observed. Commonly, the austenite grain size increased with the high-temperature holding process. For SSCP-HQT, quenching at 850 °C and at tempering 500 °C were the temperature ranges for intergranular sensitization of 304 SS, within which C atoms combined with Cr atoms to form chromium carbide precipitation. For SSCP-HST, carbides were dissolved during solution at 1150 °C, and subsequent tempering at 500 °C produced fine and uniformly distributed carbide particles. Hence, more chromium carbides are shown in Figure 2b compared to Figure 2d.

**Figure 3:**
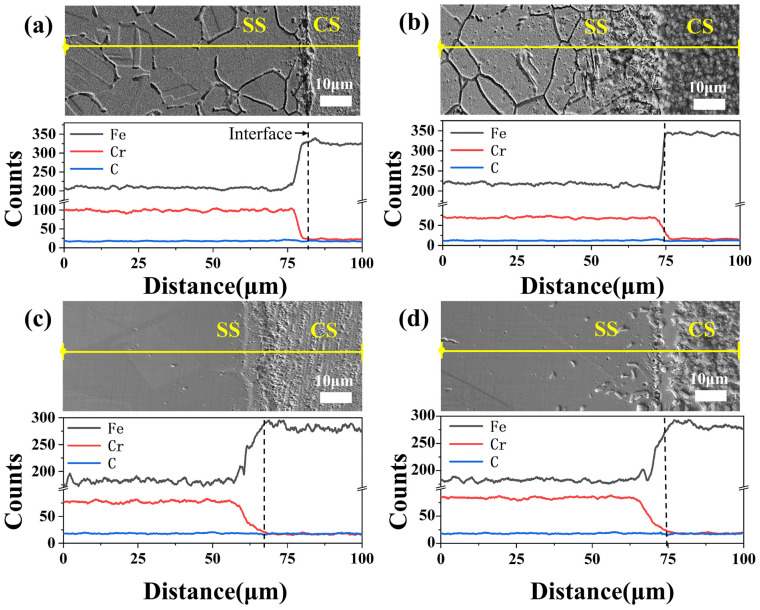
Line scanning near the bonding joint of specimens: (**a**) SSCP-H, (**b**) SSCP-HQT, (**c**) SSCP-HS, (**d**) SSCP-HST.

Due to differences in the elements between the CS base and SS cladding, mutual diffusion is bound to occur. In order to investigate the interfacial element diffusion behavior in detail, line scanning near the joint was undertaken, as shown in Figure 3. The lack of Cr in CS resulted in diffusion of Cr atoms from the cladding layer to substrate. Specifically, more significant diffusion behavior and a gentler concentration gradient of Cr atoms are shown in Figure 3c,d, under the high solution treatment temperature for SSCP-HS and SSCP-HST specimens. More C atoms diffused towards SS cladding at 1150 °C. However, the greater C atom diffusion towards SS cladding did not necessarily mean that the precipitation of chromium carbides was higher. By comparing changes in the carbide distribution of the specimens, it can be found that carbide of SSCP-HQT had a relatively large difference at the joint, indicating that more carbide was generated from SS cladding. This phenomenon was somewhat reduced in SSCP-H. Yet microstructure evolution was almost absent in SSCP-HS and SSCP-HST, suggesting relatively minor carbide formation at this time. This was because the precipitation of chromium carbide occurred within its sensitization temperature range (400–850 °C), while 1150 °C was within the solid solution temperature range of 304 stainless steel. Chromium carbide underwent dissolution and was dissolved back into the austenite grains as a solid, and the rapid cooling process prevented the precipitation of chromium carbide.

**Figure 4:**
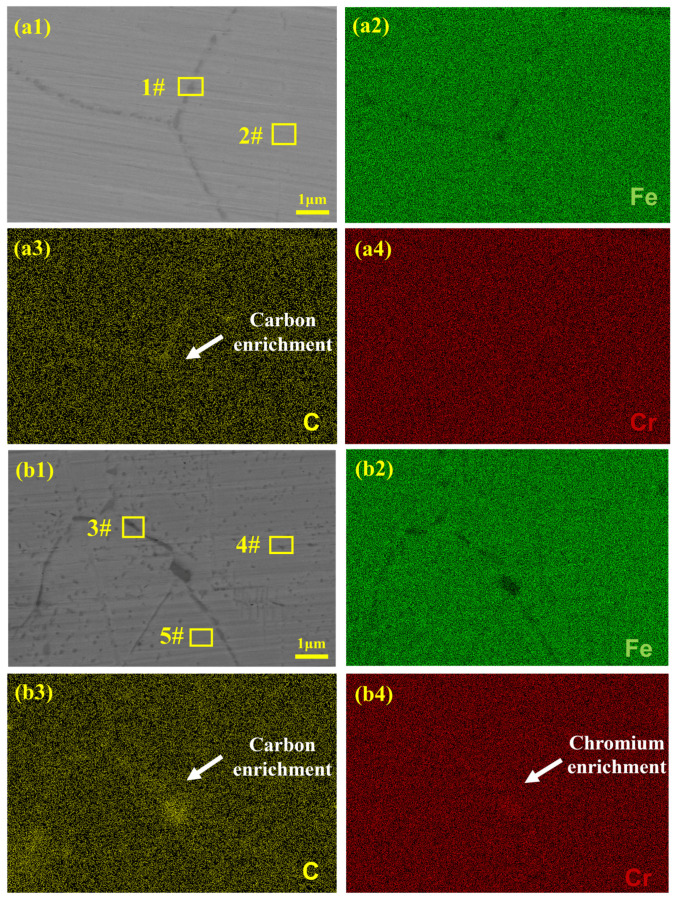
SEM images of grain boundaries at the SS cladding’s outside surface: (**a1**) SSCP-H, corresponding EDS elemental mappings of (**a2**) Fe, (**a3**) C, and (**a4**) Cr, (**b1**) SSCP-HQT, corresponding EDS elemental mappings of (**b2**) Fe, (**b3**) C, and (**b4**) Cr.

To examine the element distribution at grain boundaries of the SS cladding’s outside surface, mapping was further employed. The SEM image of SSCP-H is shown in Figure 4(a1). Grain boundaries can be clearly identified, which were the result of a large number of precipitations at grain boundaries. This was consistent with the conventional austenitic grain boundary sensitization mechanism of IGC [19,20]. As can be seen from Figure 4(a2–a4), the distribution of C and Fe within and outside grain boundary was uneven; C was enriched at the grain boundary, where Fe showed a deficit state. However, there was no significant enrichment or deletion in the distribution of Cr.

The SEM image of SSCP-HQT is exhibited in Figure 4(b1). It can be observed that in addition to grain boundaries, there were also a large number of precipitates within grains. As can be seen in Figure 4(b2–b4), the contents of C and Cr atoms were elevated in SSCP-HQT, which was similar to the rise of carbide. In addition, enrichment of C and Cr atoms also caused segregation, indicating chromium carbide precipitation, which was consistent with the typical element distribution of Cr-rich carbides at grain boundaries [21]. On the one hand, quenching at 850 °C and tempering at 500 °C were within the sensitization temperature range of stainless steel, and C atoms were prone to combine with Cr atoms, forming chromium carbide. On the other hand, the tempering process increased the diffusion of elemental atoms between the carbon steel base and stainless cladding, and, in particular, further increased the content of the element C in stainless steel, resulting in more precipitation. This phenomenon led to more serious intergranular corrosion of SS cladding.

According to existing research, the main reason for IGC is the formation of harmful secondary phases [18]. Combined with above metallographic results, precipitates were eroded away, resulting in corrosive pits. The traditional sensitization mechanism generally assumes that austenitic stainless steel IGC is caused by precipitation of Cr-rich carbides at grain boundaries and the appearance of chromium-poor areas [22,23]. The results of EDS analysis conducted to determine precipitate compositions in SSCP-H and SSCP-HQT are shown in Table 3. Precipitates at grain boundaries (1#) and within grains (2#) were detected. As a result, a high content of C (32.7%) and Cr (35.0%) was detected at grain boundaries, implying the main precipitation phase of chromium carbide, which had been proved by the findings of Ding et al. [24]. EDS tests were then conducted on grain boundary precipitates (3#), intragranular precipitates (4#), and blank areas in grains (5#). Both intragranular and intergranular precipitates possessed high contents of C and Cr, where there was a significant decrease in C (18.0%) and Cr (15.1%) contents in intragranular blanks. In summary, it can be judged that both the grain boundaries and the precipitated phase within grains are chromium carbides.

### 3.2. Mechanical Properties and Fracture Characteristics

#### 3.2.1. Microhardness of Bonding Joints

**Figure 5:**
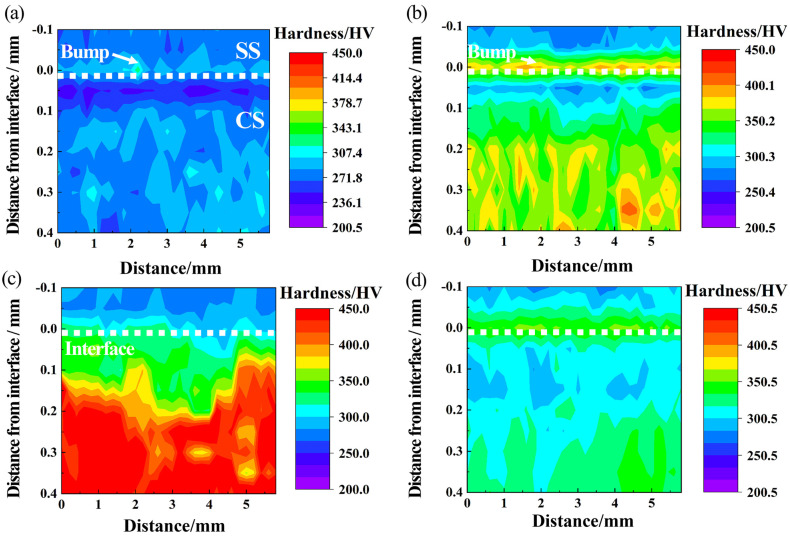
Microhardness distribution of bonding joints: (**a**) SSCP-H, (**b**) SSCP-HQT, (**c**) SSCP-HS, (**d**) SSCP-HST.

Figure 5 plots microhardness variation in bonding joints with heat treatment conditions [25,26]. The position of the bonded interface was selected as a null point. An increase in the microhardness value from the SS surface to the bonding interface is presented. SSCP-H (Figure 5a) had a bump, which was caused by C diffusion during the hot-rolling process. After heat treatment, microhardness values increased because of further diffusion and aggregation of C atoms in SS cladding. The hardness in SSCP-HQT (Figure 5b) near the bonding surface of SS cladding was highest due to a large amount of Cr_23_C_6_ precipitation, followed by dispersion strengthening. Microhardness on the CS base near the bonding interface dropped sharply because of the formation of a decarburization layer. Subsequently, microhardness increased gradually and tended to stabilize with increasing distance from the bonding joint. The microhardness value in SSCP-HS was enhanced because the strengthening of the solid solution played an important role. In Figure 5d, the hardness value of SSCP-HST increased due to a small amount of uniform precipitation of chromium carbide. However, the microhardness value of the CS base decreased, but it was still higher than that of SSCP-H.

#### 3.2.2. Shear Behavior and Fracture Characteristics

**Figure 6:**
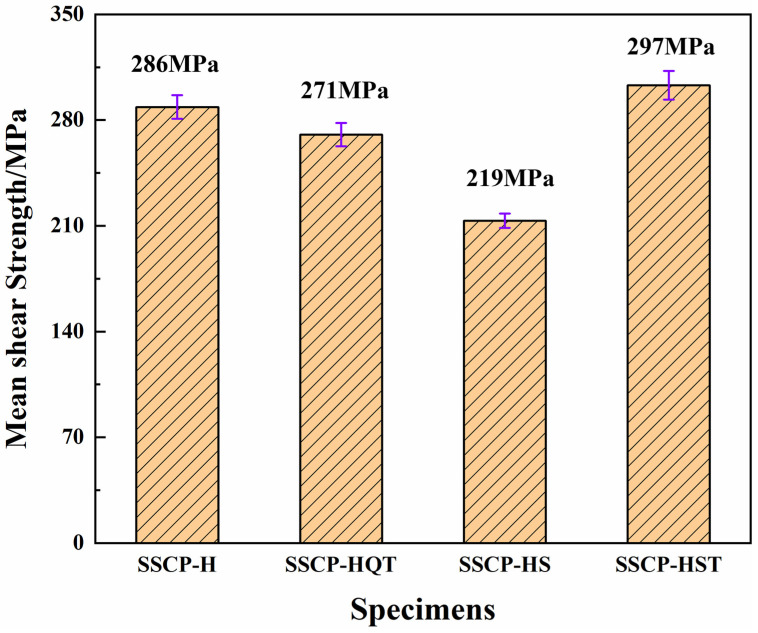
Mean shear strength of joints under different heat treatment conditions.

Joint strength is an important indicator to evaluate the performance of stainless steel cladding plates [27,28]. Therefore, a shear strength test of joints was conducted, as shown in Figure 6. The shear strength of SSCP-H was about 286 MPa. After quenching and tempering, the shear strength was lowered to 271 MPa. Serious intergranular corrosion induced by precipitation led to weak connections at grain boundaries. The shear strength of SSCP-HS and SSCP-HST was 219 MPa and 297 MPa, respectively. The shear strength obtained depended mainly on the strength of SS cladding as the fractures in the shear test were on the SS side. The precipitation amount of SSCP-HST carbide was reduced, so the shear strength was improved. Although the carbide content was reduced after solution treatment, the SS cladding was soft, so the bonding strength was the lowest. According to GB/T8165-2008, all SSCP specimens meet the sheer strength requirements. Specifically, the joint strength of SSCP could be improved by solution and tempering treatment.

**Figure 7:**
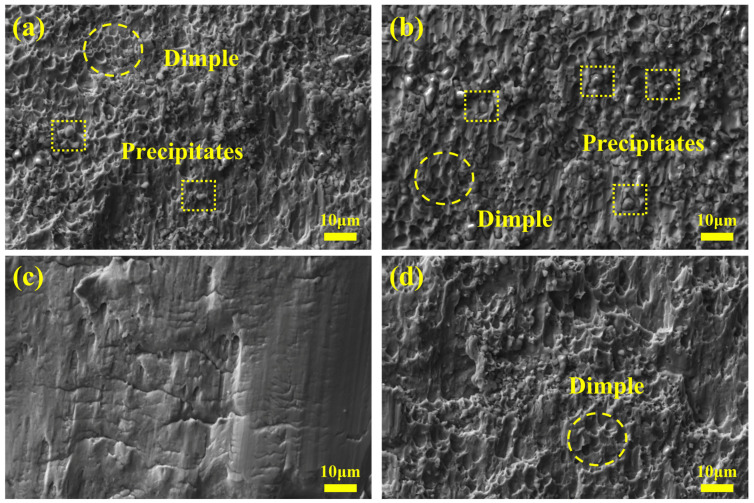
Shear fracture morphologies of SS cladding: (**a**) SSCP-H, (**b**) SSCP-HQT, (**c**) SSCP-HS, (**d**) SSCP-HST.

Shear fracture morphologies of SS cladding are shown in Figure 7. As observed in Figure 7a,b, shear fracture morphologies of SSCP-H and SSCP-HQT were typical dimple fractures, indicating they had good toughness. In addition, precipitates could be found in fracture morphologies, and precipitates in SSCP-HQT were distinct compared with those in SSCP-H. This was consistent with the analysis mentioned above. Commonly, carbide precipitated at grain boundaries after quenching and tempering can promote the rise of hardness and brittleness. As seen in Figure 7c, the fracture morphology of SSCP-HS was a pure shear fracture morphology. Constantly, SSCP-HST fracture morphology was basically a tough nest fracture, as shown in Figure 7d. As a result, SSCP-HST possessed better toughness, which was beneficial to the safe use of SSCP.

### 3.3. Electrochemical Testing

#### 3.3.1. DL-EPR Test

**Figure 8:**
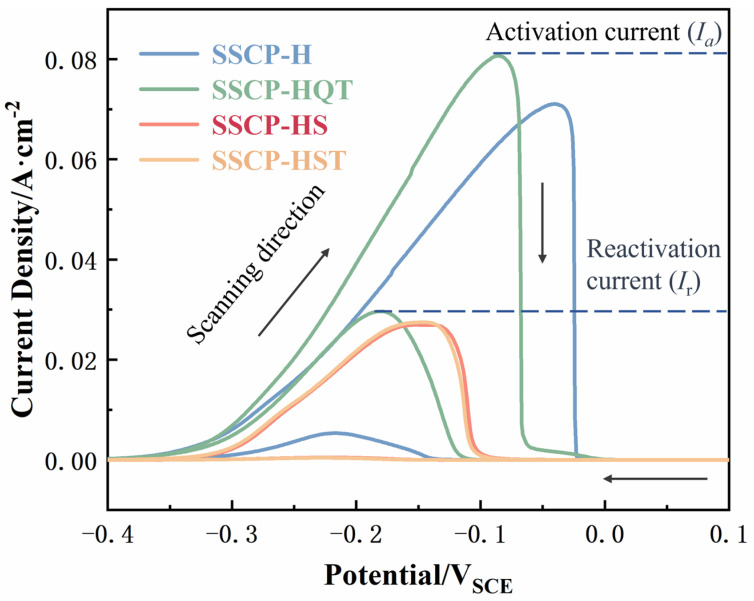
DL-EPR curves for the SS cladding surface with various heat treatment conditions.

In order to study the IGC resistance of SS cladding with heat treatment conditions, a DL-EPR test was carried out [29,30]. Results are shown in Figure 8. It was observed that current density increased with potential in the period of the anodic polarization scan loop. The maximum current density (I_a_) was reached when the potential was around −100 mV, and then dropped dramatically after passivation. With the rise in potential, the current density decreased to a relatively stable value called the passive current density. During the cathodic polarization scan loop, significant reactivation peaks could be observed in SSCP-H and SSCP-HQT. However, the reactivation bump was barely visible in curves of SSCP-HS and SSCP-HST.

Based on DL-EPR curves, values of activation current density (I_a_) and reactivation current density (I_r_) of specimens with various heat treatment conditions were obtained. The degree of sensitization (DOS) value was calculated by the ratio of I_r_/I_a_ in DL-EPR curves [31]. Table 4 reports the sensitization degree for the SS cladding surface. It can be seen that DOS values of SSCP-H and SSCP-HQT were 7.51% and 36.8%, respectively. The higher I_r_ values indicated that a certain degree of Cr-depleted region dissolution occurred, and specimens were more susceptible to IGC. As can be seen from Figure 4(b1), more carbides precipitated during the quenching and tempering treatment, which led to more dissolution of the Cr-depleted zone and a more serious IGC phenomenon. However, both DOS values of SSCP-HS and SSCP-HST were approximately 0% because of the absence of reactivation peaks. As mentioned previously, chromium carbides in SS cladding were dissolved into austenite after solution treatment. In addition, specimens after solution treatment were tempered, which did not cause enough reprecipitation of chromium carbide. The SS cladding surface still expressed excellent resistance to IGC. Again, the variation in DOS was consistent with the presence of chromium carbide precipitates.

**Figure 9:**
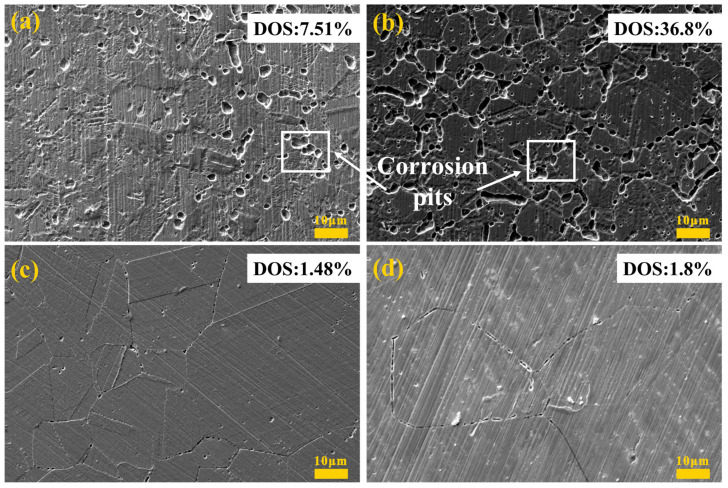
Corrosion morphology of the SS cladding surface after DL-EPR test: (**a**) SSCP-H, (**b**) SSCP-HQT, (**c**) SSCP-HS, (**d**) SSCP-HST.

Figure 9 shows the morphology of specimens with heat treatment conditions after DL-EPR tests. The distinctness of SEM morphologies was in great agreement with that of DOS values. Figure 9a shows the quantity of discontinuous corrosion pits at grain boundaries. As can be seen from Figure 9b, pits increased significantly and connected into a network structure, which might be associated with more precipitates in SSCP-HQT than in SSCP-H. However, grain boundaries are clearly visible in Figure 9c,d and there are few signs of intergranular corrosion.

#### 3.3.2. Potentiodynamic Polarization

**Figure 10:**
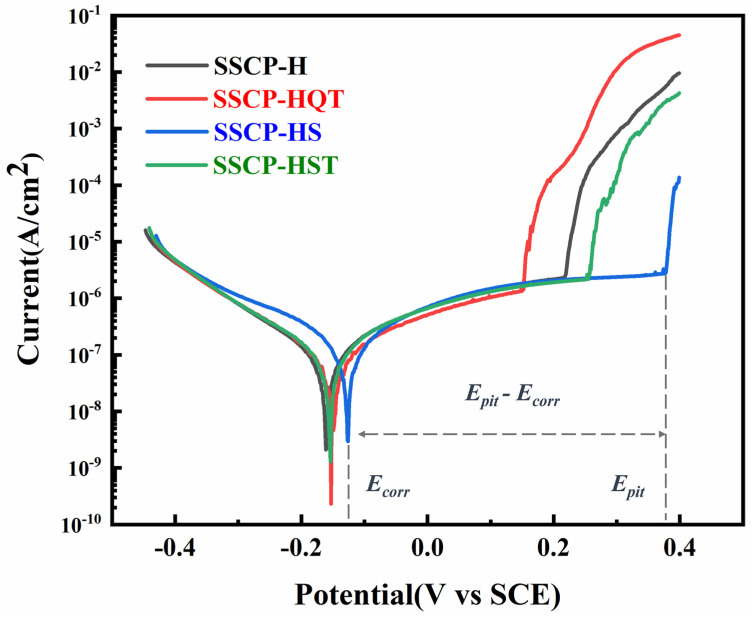
Potential polarization curves for the SS cladding surface with heat treatment conditions.

To investigate the effect of different heat treatments on the corrosion resistance of SS cladding in 3.5 wt.% NaCl solution, dynamic potential polarization tests were performed, as shown in Figure 10. Specific electrochemical parameters of corrosion potential (E_corr_) and pitting potential (E_pit_) are listed in Table 5 [32]. It was noted that the passivation interval (|E_pit_ − E_corr_|) and pitting potential of the SS cladding surface differed significantly with heat treatment conditions. As carbide content increased, E_pit_ and the potential range of the passivation zone gradually decreased. In comparison, it can be found that SSCP-HQT had the lowest E_pit_ (0.154 V), which was lower than that of SSCP-H (0.219 V) and SSCP-HS (0.376 V). In conclusion, pitting resistance had a significant relationship with carbide precipitation.

According to the above results, it can be found that intergranular sensitivity accompanied by the precipitation of carbide played a key role in promoting pitting corrosion behavior. The formation of chromium carbide led to the absence of Cr in the Cr-depleted zone. However, the passive film in the SS anticorrosion mechanism was precisely due to the presence of Cr. With the increase in chromium carbide precipitation, a Cr-depleted zone appeared. There were fewer Cr elements used to form the passive film, so the pitting corrosion resistance decreased. Conversely, chromium carbide precipitates disappeared after solution treatment, and SS cladding had the ability to form a complete passive film. Therefore, it had a high pitting potential and excellent resistance to pitting corrosion.

#### 3.3.3. Electrochemical Impedance Spectroscopy

**Figure 11:**
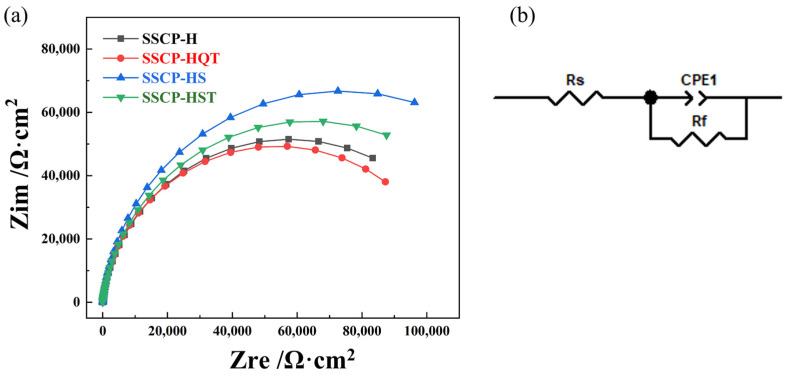
EIS test results for the SS cladding surface with heat treatment conditions: (**a**) Nyquist plot; (**b**) equivalent circuit.

To evaluate the corrosion resistance mechanism of SSCP via different heat treatments, the Nyquist plot of EIS test results were drawn, as shown in Figure 11a. Figure 11b showed the equivalent circuit for impedance fitting. Electrochemical parameters were shown in Table 6, where Rs was solution resistance, Rf was charge transfer resistance, and CPE was double-layer capacitance. The fitting results were similar to the experimental values with the [R(QR)] circuit, illustrating that the selected fitting circuit can accurately describe the impedance behavior [33]. As shown in Figure 11a, Nyquist plots were all composed of a single semicircular reactance arc. In general, the larger the radius of the curve, the better the polarization resistance and corrosion resistance of alloys. Additionally, the capacitance arc radius decreased gradually as carbide content increased. This indicates that the passive film formed on the surface of the specimen was unstable, and the protective effect of the SS cladding passivation film gradually decreased with the rise of carbide. SSCP-HS had the largest radius of curvature, indicating the noblest polarization electron resistance and corrosion resistance.

## 4. Conclusions

(1) Diffusion of carbon atoms from the CS base to SS cladding might be promoted by hot rolling and heat treatment as long as it is in range of austenite intergranular sensitization. This affects the mechanical properties of bonding joints and corrosion resistance of SS cladding.
(2) Stainless steel cladding of SSCP-H and SSCP-HQT exhibited different degrees of intergranular sensitization. However, almost no intergranular sensitization zone was observed in SSCP-HS and SSCPP-HST.
(3) The bonding strength between CS and SS was closely associated with precipitation. Solution and tempering treatment with a proper temperature played a critical role in promoting carbide dissolution and retaining the carbon atoms’ reinforcement in the joints of SSCP, so the shear strength of SSCP-HST increased to 297 MPa.
(4) SSCP-HS showed the lowest corrosion sensitization due to the dissolution of carbide in austenite. Specifically, tempering after solution treatment did not cause enough reprecipitation of chromium carbide. The DOS of SSCP-HST was 1.80 % and the value of E_pit_ was 247 mV, which indicated that SSCP-HST still had good corrosion resistance.
(5) SSCP-HST not only exhibited good mechanical properties of the CS base, but also had excellent corrosion resistance of the SS cladding. The comprehensive performance of SSCP was improved.

## Figures and Tables

**Table 1:** Chemical compositions of stainless steel and carbon steel in SSCP (wt. %).

Material	Elements					
	C	Si	Mn	Ni	Cr	Fe
304 stainless steel	0.05	0.44	1.21	8.13	18.28	Bal.
45 carbon steel	0.43	0.35	0.55	0.16	0.21	Bal.

**Table 2:** Heat treatment processes of different specimens.

Specimen	Processing Conditions
SSCP-H	Hot rolling
SSCP-HQT	Hot rolling + Quenching (at 850 °C for 0.5 h, WC) + Tempering (at 500 °C for 1 h, AC)
SSCP-HS	Hot rolling + Solution (at 1150 °C for 0.5 h, WC)
SSCP-HST	Hot rolling + Solution (at 1150 °C for 0.5 h, WC) + Tempering (at 500 °C for 1 h, AC)

**Table 3:** EDS analysis results of different areas in SEM images.

Element	C (at%)	Cr (at%)	Fe (at%)
1#	35.0	32.7	28.7
2#	15.4	16.7	59.3
3#	33.5	44.7	20.9
4#	29.6	33.6	33.6
5#	15.1	18.0	58.1

**Table 4:** Results of DOS for the SS cladding surface with heat treatment conditions.

Specimen	I_a_ (A cm^−2^)	I_r_ (A cm^−2^)	DOS (I_r_/I_a_ × 100%)
SSCP-H	7.10 × 10^−2^	5.33 × 10^−3^	7.51
SSCP-HQT	8.07 × 10^−2^	2.97 × 10^−2^	36.80
SSCP-HS	2.75 × 10^−2^	4.08 × 10^−4^	1.48
SSCP-HST	2.69 × 10^−2^	4.84 × 10^−4^	1.80

**Table 5:** Results of potential polarization curves for the SS cladding surface with heat treatment conditions.

Specimen	E_corr_ (V)	E_pit_ (V)	|E_pit_ − E_corr_| (V)
SSCP-H	−0.161	0.219	0.380
SSCP-HQT	−0.153	0.154	0.307
SSCP-HS	−0.126	0.376	0.502
SSCP-HST	−0.154	0.247	0.401

**Table 6:** Equivalent circuit fitting parameters of the SS cladding surface with heat treatment conditions.

Specimen	Rs (Ω·cm^2^)	Rf (Ω·cm^2^)	CPE (S·s^n^·cm^−2^)	n
SSCP-H	6.89	1.15 × 10^5^	5.68 × 10^−5^	0.927
SSCP-HQT	6.39	1.08 × 10^5^	4.89 × 10^−5^	0.942
SSCP-HS	6.04	1.47 × 10^5^	5.51 × 10^−5^	0.938
SSCP-HST	6.59	1.26 × 10^5^	5.75 × 10^−5^	0.921

## Data Availability

Not applicable.
